# Non-coding transcriptomic profiles in the sheep mammary gland during different lactation periods

**DOI:** 10.3389/fvets.2022.983562

**Published:** 2022-11-08

**Authors:** Weihao Chen, Xinyu Gu, Xiaoyang Lv, Xiukai Cao, Zehu Yuan, Shanhe Wang, Wei Sun

**Affiliations:** ^1^College of Animal Science and Technology, Yangzhou University, Yangzhou, China; ^2^Joint International Research Laboratory of Agriculture and Agri-Product Safety of Ministry of Education of China, Yangzhou University, Yangzhou, China; ^3^International Joint Research Laboratory in Universities of Jiangsu Province of China for Domestic Animal Germplasm Resources and Genetic Improvement, Yangzhou University, Yangzhou, China

**Keywords:** sheep, mammary gland, lactation, circRNA, miRNA, machine learning, ceRNA

## Abstract

Sheep milk production is a dynamic and multifactorial trait regulated by diverse biological mechanisms. To improve the quality and production of sheep milk, it is necessary to understand the underlying non-coding transcriptomic mechanisms. In this study, ribonucleic acid-sequencing (RNA-seq) was used to profile the expression of microRNAs (miRNAs) and circular RNAs (circRNAs) in the sheep mammary gland at three key lactation time points (perinatal period, PP; early lactation, EL; and peak lactation, PL). A total of 2,369 novel circRNAs and 272 miRNAs were profiled, of which 348, 373, and 36 differentially expressed (DE) circRNAs and 30, 34, and 7 DE miRNAs were detected in the comparison of EL vs. PP, PL vs. PP, and PL vs. EL, respectively. A series of bioinformatics analyses including functional enrichment, machine learning prediction, and competing endogenous RNA (ceRNA) network analyses were conducted to identify subsets of the potential candidate miRNAs (e.g., oar_miR_148a, oar_miR_362, and oar_miR_432) and circRNAs (e.g., novel_circ_0011066, novel_circ_0010460, and novel_circ_0006589) involved in sheep mammary gland development. Taken together, this study offers a window into the dynamics of non-coding transcriptomes that occur during sheep lactation and may provide further insights into miRNA and circRNA that influence sheep mammary gland development.

## Introduction

Sheep have been used to supply dairy products for centuries and rank fourth in global milk production (1). For these reasons, increasing the yield and quality of sheep milk is a desirable goal in the dairy sheep industry. However, milk production is a dynamic and multifactorial trait regulated by diverse molecular mechanisms and has a moderate heritability (2), which emphasizes the importance of precise selection for sheep milk production.

With the development of nucleic acid sequencing technologies, high-throughput sequencing has allowed for in-depth investigation of coding and non-coding transcripts in the lactating mammary gland of dairy species (3–5). In a previous study (6), we profiled the expression of messenger ribonucleic acids (mRNAs) and long non-coding RNAs (lncRNAs) in the sheep mammary gland during different lactation periods. However, due to the lack of information on non-coding RNAs unrelated to lncRNAs, such as microRNAs (miRNAs) and circular RNAs (circRNAs), the study only provided a partial view of the transcriptomic profile of the sheep mammary gland.

Circular RNA, a recently discovered non-coding RNA, has received considerable attention in mammary gland research. Xu et al. (7) identified a significantly greater number of circRNAs in the human mammary gland than in other tissues such as adrenal glands and thyroid, and similar results were found in sheep (3), cattle (8), and rats (9), indicating the vital roles of circRNAs in the mammary glands of various species. Moreover, some circRNAs that share miRNA recognition elements with miRNA target genes can enhance the expression of those genes by acting as miRNA sponges (10), initially modulating the cross talk between circRNA, miRNA, and its target genes in what is referred to as a “competitive endogenous RNA interaction” (ceRNA). Circ003429 enhances the expression of *YAP1* by sponging miR-199a-3p during fatty acid synthesis in dairy goats (11). In dairy cows, circ11103 regulates milk fat metabolism *via* the miR-128/PPARGC1A axis (12). Collectively, these studies highlight the coordinated regulation of mammary gland development by circRNAs and miRNAs. However, the specific roles of these molecules remain largely unknown, especially in dairy sheep.

Numerous studies have well investigated the roles of circRNAs (8, 13) and miRNAs (14) in mammary gland development in various species, based on the universal characteristics of circRNAs and miRNAs and their potential ceRNA regulation in mammary glands. However, few reports have described dynamic RNA expression profiles or associated mechanisms in the sheep mammary gland during different lactation periods.

The mammary gland is a key organ related to lactation in mammals, and milk yield is largely controlled by mammary epithelial cells (MECs). From the beginning of pregnancy to the end of the perinatal period (PP), the mammary gland develops further and a rapid proliferation of MECs takes place (15). After parturition, MECs differentiate into secretory cells, regulate lactation, and remain stable during lactation. During peak lactation (PL) to late lactation, milk yield begins to decrease and apoptosis of MECs begins (16). Hence, we selected three key time points in the development of MECs to study the molecular mechanisms underlying sheep lactation: perinatal period, early lactation (EL), and PL. In this study, RNA-seq was used to profile the expression of miRNAs and circRNAs in the sheep mammary gland at three key lactation points. A series of bioinformatics and machine learning approaches were used to identify key circRNAs and miRNAs involved in mammary gland development, and a network of ceRNAs was constructed to better understand their roles in sheep lactation.

## Materials and methods

### Sample collection

All experimental sheep were supplied by Zhenjiang Wan Shan Hong Bian Agricultural Co., Ltd. (Zhenjiang, Jiangsu province, China). Detailed information on the experimental sheep can be found in our previous report (6).

Briefly, mammary gland biopsy tissues were collected from first-time pregnant Hu ewes with similar pregnancy dates and litter size, at three important lactation periods: 5 days before expected parturition (perinatal period, PP), 6 days postpartum (EL), and 25 days postpartum (PL). The collected mammary gland biopsy tissues were snap-frozen in liquid nitrogen and stored at −80°C before RNA extraction.

### RNA extraction and sequencing

Ribonucleic acid (RNA) was extracted from the stored mammary gland biopsy tissues with the TRIzol reagent (Invitrogen, Carlsbad, CA, USA). The quality and integrity of the isolated RNA were examined with an RNA Nano 6000 Assay kit and Agilent 2100 Bioanalyzer, respectively.

MicroRNAs libraries were constructed with the NEB Next^®^ Multiplex Small RNA Library Prep Set for Illumina^®^ (NEB, Ipswich, MA, USA). circRNA libraries were constructed with the NEBNext^®^ Ultra™ Directional RNA Library Prep kit for Illumina^®^ (NEB). The miRNA and circRNA libraries were sequenced on the Illumina HiSeq^TM^ 2500 platform (a single-end 50 bp strategy and a paired-end 150 bp strategy for miRNA and circRNA sequences, respectively) by Beijing Novogene Technology Co., Ltd. (Beijing, China).

Raw reads were generated in the FASTQ format, and reads containing poly-N, adapters, or poly A, T, C, or G and low-quality reads were removed by fastp (17). The clean reads obtained were mapped to the *Ovis aries* reference genome (Oar_v4.0) using Hisat2 (18). miRbase 20.0, as a known miRNA alignment, was used as a reference, miRDeep2 (19) was used to assemble miRNA transcripts, and srna-tools-cli was used to identify the potential miRNAs and draw their secondary structure. miREvo (20) and miRDeep2 were used to distinguish novel miRNA candidates from the transcripts by examining their secondary structure. circRNA candidates were distinguished from transcripts with find_circ (21) and CIRI2 (22), and detailed parameters used for the aforementioned software are provided in Supplementary Table S1.

### Differentially expressed transcripts

The transcripts per million (TPM) parameter was used to estimate the expression levels of miRNA and circRNA transcripts. Multiple comparisons were used to identify differentially expressed (DE) circRNAs and miRNAs among the PP, EL, and PL groups using the DEseq R library (23). Transcripts were deemed significantly differentially expressed (DE) when the threshold of the adjusted *p*-value (adjusted *p*-value with the false discovery rate (FDR) approach) was < 0.05.

### Gene ontology and Kyoto encyclopedia of genes and genomes enrichment

The target genes of DE miRNAs (predicted with miRanda and RNAhybird) and the parental genes of DE circRNAs were functionally annotated. The GOseq R library (24) and KO-Based Annotation System (KOBAS) (25) were used to determine gene ontology (GO) and Kyoto Encyclopedia of Genes and Genomes **(**KEGG) enrichment, respectively. Fisher's exact test with the FDR multiple test correction was used to assess statistical significance (*p* < 0.01), and the detailed parameters used for GOseq and KOBAS are provided in Supplementary Table S1.

### Investigation of sheep lactation biomarkers using machine learning approaches

To identify non-coding RNA biomarkers for predicting sheep lactation, a two-step decision tree machine learning method entitled Random Forest-XGBoost (RX) was used. The R library randomForest (RF) and XGBoost were used for analysis. A detailed strategy for RX was described in our previous study (26).

In brief, a range of parameters (Ntree and mtry for RF and colsample and eta for XGBoost) was systematically evaluated by examining the out-of-bag (OOB) error rate to determine the derive minimum hyperparameter values required for RF and XGBoost.

To identify the biomarkers of sheep lactation, all samples were divided into three classes according to lactation stage (PP, EL, or PL). The miRNA and circRNA expression data sets were first offered to Random Forest to select variables (miRNAs and circRNAs) with positive values for their variable importance measures (VIMs). The positive-VIM subset of variables was then assessed with XGBoost. Similarly, XGBoost generated VIMs for the variables designated “Gain.”

The VIM value of an individual variable (circRNA or miRNA) denotes the relative contribution of the variable to each decision tree; the higher the VIM value, the more important the variable is to distinguish different classes (sheep lactation stages). Therefore, variables with a high “Gain” were prioritized as the potential non-coding RNA biomarkers of sheep lactation.

### ceRNA network construction

Messenger RNA (mRNA) expression data sets for the sheep mammary gland during different lactation stages were obtained in our previous study and are available on: https://www.ncbi.nlm.nih.gov/, PRJNA759095. First, miRanda (27), and RNAhybrid (28) were used to predict miRNA-binding seed sequence sites and target mRNAs or circRNAs, detailed parameters for miRanda and RNAhybrid are provided in Supplementary Table S1. The miRNA–mRNA and miRNA–circRNA interaction pairs that shared the same miRNAs were then selected for subsequent analysis as candidate competing endogenous interactions. Pearson's correlation coefficients (PCCs) and corrected *p-*value (adjusted with Benjamini and Hochberg's approach) were calculated for the expression of the candidate circRNAs, miRNAs, and mRNAs. Finally, negatively regulated miRNA–mRNA/circRNA pairs with PCC < −0.75 and a corrected *p* < 0.05 were selected to establish ceRNA networks with the Cytoscape software (29).

### Sequencing data validation

Five miRNAs and circRNAs were randomly chosen for the validation of the RNA-seq data, *GAPDH* and U6 were selected as the reference gene, and the primers were designed with Primer Premier 5 software (Supplementary Table S2).

Total RNA was extracted from the mammary gland biopsy samples with the TRIzol reagent according to the manufacturer's instructions. The extracted RNA was then reverse transcribed into complementary DNA (cDNA) with FastKing gDNA Dispelling RT SuperMix (Vazyme Biotech, Nanjing, Jiangsu, China), according to the manufacturer's instructions.

Real-time quantitative polymerase chain reaction (PCR) was performed in triplicate with cDNA as a template. The 2^−Δ*ΔCt*^ method (30) was used to calculate relative expression levels. The results were presented as fold changes in relative expression levels, using the GraphPad Prism 6 software.

## Results

### An overview of the sequencing data

In the miRNA libraries, the average rates of clean reads were 96.54% (EL), 96.03% (PL), and 96.41% (PP), and the average mapping rates were 95.71% (EL), 96.17% (PL), and 95.12% (PP). In the circRNA libraries, the average rates of clean reads were 97.23% (EL), 96.98% (PL), and 97.68% (PP), and the average mapping rates were 83.23% (EL), 80.03% (PL), and 81.71% (PP). Detailed information on the circRNA and miRNA libraries is given in Supplementary Table S3.

**Figure 1:**
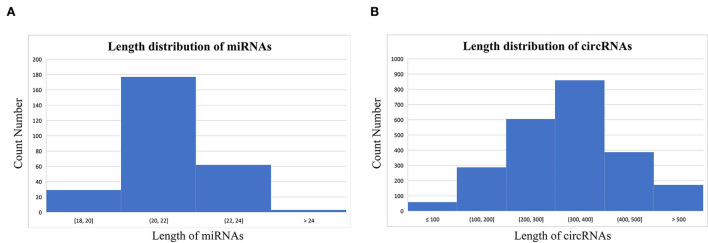
Length distribution of the distinguished micro ribonucleic acids (miRNAs) **(A)** and circular RNAs (circRNAs) **(B)**.

Based on the results of miREvo, miRDeep2, find_circ, and CIRI2, a total of 2,369 novel circRNAs and 272 miRNAs (140 annotated miRNAs and 132 novel miRNAs) were identified. The majority of circRNAs were 200–400 nt long, whereas the majority of miRNAs were 20–24 nt long. The average length of circRNAs was 326.69 nt, whereas miRNAs had an average length of 21.76 nt (Figure 1).

### Expression profiles of miRNAs and circRNAs

Transcripts per million was used to normalize the expression of miRNA and circRNA transcripts, based on which DEseq was used to identify DE miRNAs and DE circRNAs among the PP, EL, and PL groups. Detailed information on the miRNA and circRNA is given in Supplementary Table S4, and the results of DE analysis are given in Supplementary Table S5. Pearson's correction between the individual samples are shown in Supplementary Figure S1.

**Figure 2:**
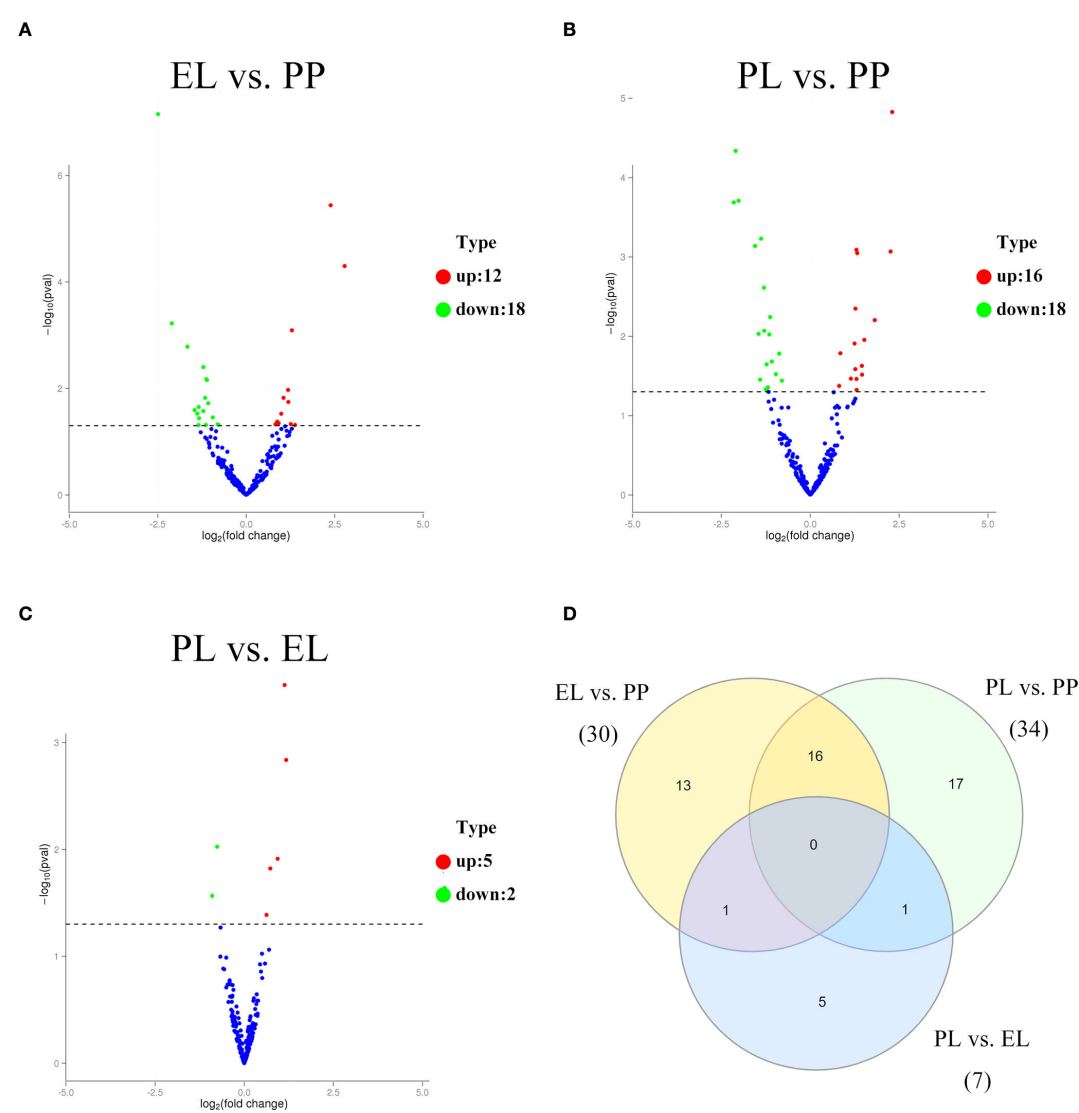
The volcano plot of differentially expressed (DE) miRNAs identified in early lactation (EL) vs. perinatal period (PP) **(A)**, peak lactation (PL) vs. PP **(B)**, and PL vs. EL comparisons **(C)**. A Venn diagram of DE miRNAs in all three comparisons **(D)**.

Of these DE miRNAs, 30, 34, and 7 were detected in the comparison of EL vs. PP (Figure 2A), PL vs. PP (Figure 2B), and PL vs. EL (Figure 2C), respectively. No miRNA was DE in all three comparisons (Figure 2D). Based on the adjusted *p*-value, the top three most DE miRNAs were oar_miR_370_3p, oar_miR_148a, and novel_miR_175 in the comparison of EL vs. PP. In the comparison of PL vs. PP, the top three most DE miRNAs were oar_miR_148a, oar_miR_370_3p, and oar_miR_99a. In the comparison of PL vs. EL, the top three most DE miRNAs were oar-miR-218a, oar-miR-3959-3p, and oar-miR-181a.

**Figure 3:**
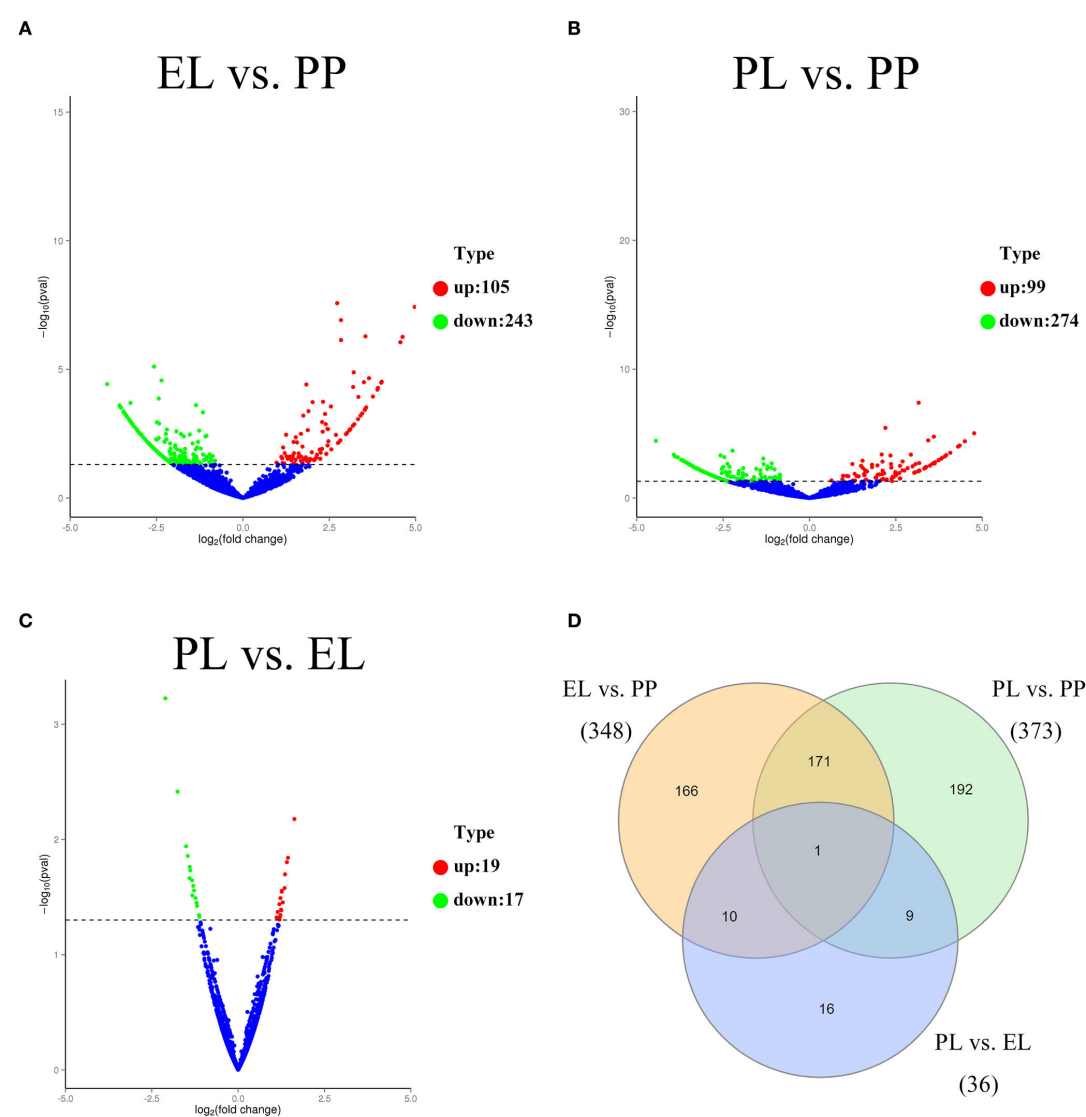
The volcano plot of DE circRNAs identified in the EL vs. PP **(A)**, PL vs. PP **(B)**, and PL vs. EL comparisons **(C)**. A Venn diagram of DE circRNAs in all three comparisons **(D)**.

Of the DE circRNAs, 348, 373, and 36 DE circRNAs were detected in the comparison of EL vs. PP (Figure 3A), PL vs. PP (Figure 3B), and PL vs. EL (Figure 3C), respectively. A Venn diagram of DE circRNAs in different comparison groups showed that novel_circ_0010160 was DE in all three comparisons (Figure 3D).

Based on the adjusted *p*-value, the top three most DE circRNAs were novel_circ_0010649, novel_circ_0010160, and novel_circ_0001655 in the comparison of EL vs. PP. In the comparison of PL vs. PP, the top three most DE circRNAs were novel_circ_0010252, novel_circ_0010649, and novel_circ_0010642. In the comparison of PL vs. EL, the top three most DE circRNAs were novel_circ_0000578, novel_circ_0000885, and novel_circ_0001489.

**Figure 4:**
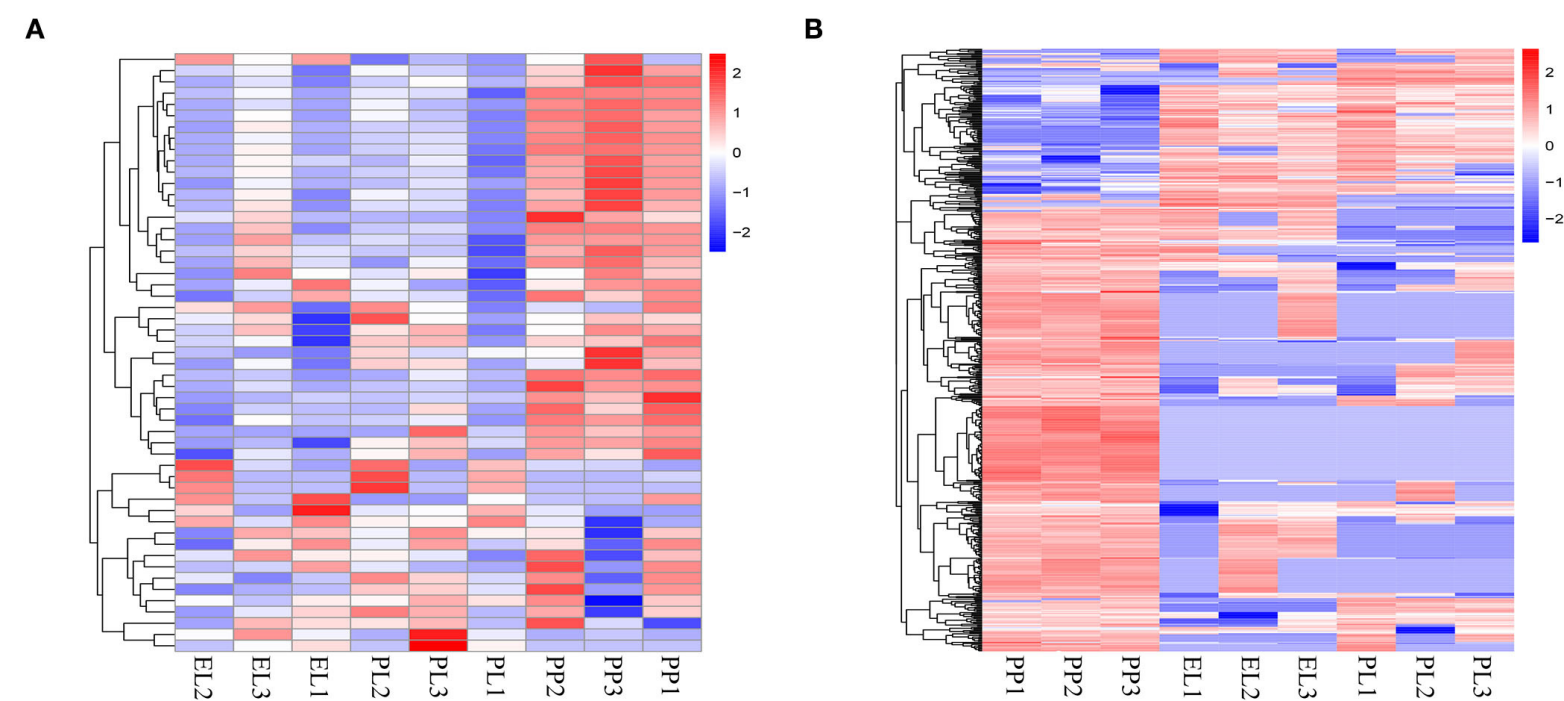
The heatmap of DE miRNAs **(A)** and DE circRNAs **(B)**.

Heat maps of DE miRNAs (Figure 4A) and DE circRNAs (Figure 4B) indicated clearly different non-coding transcriptomic profiles in the non-lactation period (PP) and the lactation period (EL and PL), but there was no obvious difference between the profiles of EL and PL.

### GO and KEGG enrichment

Gene ontology and KEGG enrichment analyses of the target genes of DE miRNAs and the parental genes of DE circRNAs were performed. These results are given in Supplementary Table S6.

**Figure 5:**
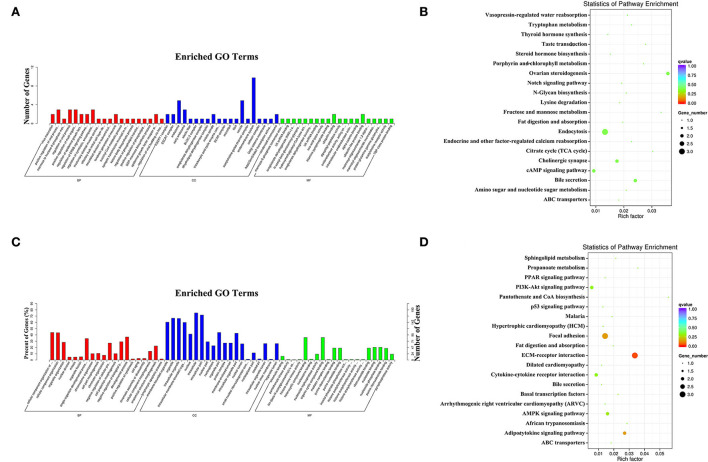
The most enriched gene ontology (GO) terms **(A)** and Kyoto Encyclopedia of Genes and Genomes (KEGG) pathways **(B)** of the target genes of the DE miRNAs identified in the EL vs. PP comparison. The most enriched GO terms **(C)** and KEGG pathways **(D)** of the parental genes of the DE circRNAs identified in the EL vs. PP comparison.

In the comparison of EL vs. PP, the target genes of the DE miRNAs were significantly enriched in 33 GO terms (Figure 5A), and the most enriched GO terms were virus maturation (GO: 0019075) in a biological process (BP), ESCRT I complex (GO: 0000813) in a cellular component (CC), and mannose-6-phosphate isomerase activity (GO: 0004476) in the molecular function (MF). The parental genes of the DE circRNAs were significantly enriched in 127 GO terms, and the most enriched GO terms were mitotic CC organization or biogenesis (GO: 0071840) in BP, membrane-bounded organelle (GO: 0043227) in MF, and chromatin binding (GO: 0003682) in MF (Figure 5C).

In the KEGG enrichment analysis, the target genes of the DE miRNAs were significantly enriched in four KEGG pathways (Figure 5B), the three most enriched KEGG pathways were ovarian steroidogenesis (oas04913), bile secretion (oas04976), and endocytosis (oas04144). The parental genes of the DE circRNAs were significantly enriched in five KEGG pathways (Figure 5D), the top three enriched KEGG pathways were ECM–receptor interaction (oas04512), focal adhesion (oas04510), and adipocytokine signaling pathway (oas04920).

**Figure 6:**
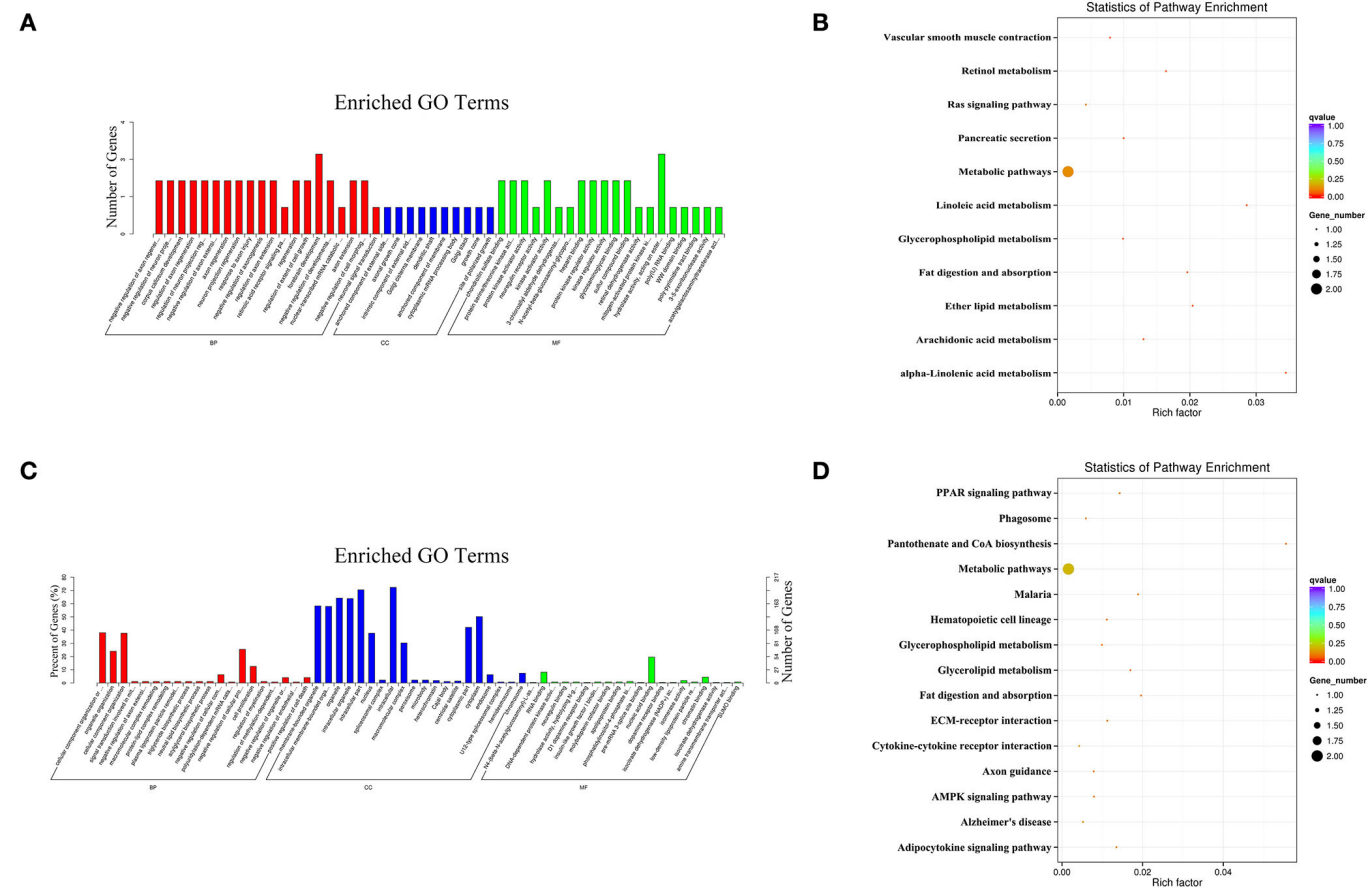
The most enriched GO terms **(A)** and KEGG pathways **(B)** of the target genes of the DE miRNAs identified in PL vs. PP comparison. The most enriched GO terms **(C)** and KEGG pathways **(D)** of the parental genes of the DE circRNAs identified in the PL vs. PP comparison.

In the PL vs. PP comparison, the target genes of the DE miRNAs were significantly enriched in 85 GO terms, and the most enriched GO terms were negative regulation of axon regeneration (GO: 0048681) in BP, anchored component of the external side of plasma membrane (GO: 0031362) in CC, and chondroitin sulfate binding (GO: 0035374) in MF (Figure 6A). The parental genes of the DE circRNAs were significantly enriched in 72 GO terms, and the most enriched GO terms were CC organization or biogenesis (GO: 0071840) in BP, membrane-bounded organelle (GO: 0043227) in CC, and N4-(beta-N-acetylglucosaminyl)-L-asparaginase activity (GO: 0003948) in MF (Figure 6C).

In terms of KEGG enrichment, the target genes of the DE miRNAs were significantly enriched in eight KEGG pathways (Figure 6B), and the three most enriched KEGG pathways were alpha-linolenic acid metabolism (oas00592), linoleic acid metabolism (oas00591), and ether lipid metabolism (oas00565). The parental genes of the DE circRNAs were significantly enriched in six KEGG pathways (Figure 6D), and the three most enriched KEGG pathways were pantothenate and CoA biosynthesis (oas00770), fat digestion and absorption (oas04975), and malaria (oas05144).

**Figure 7:**
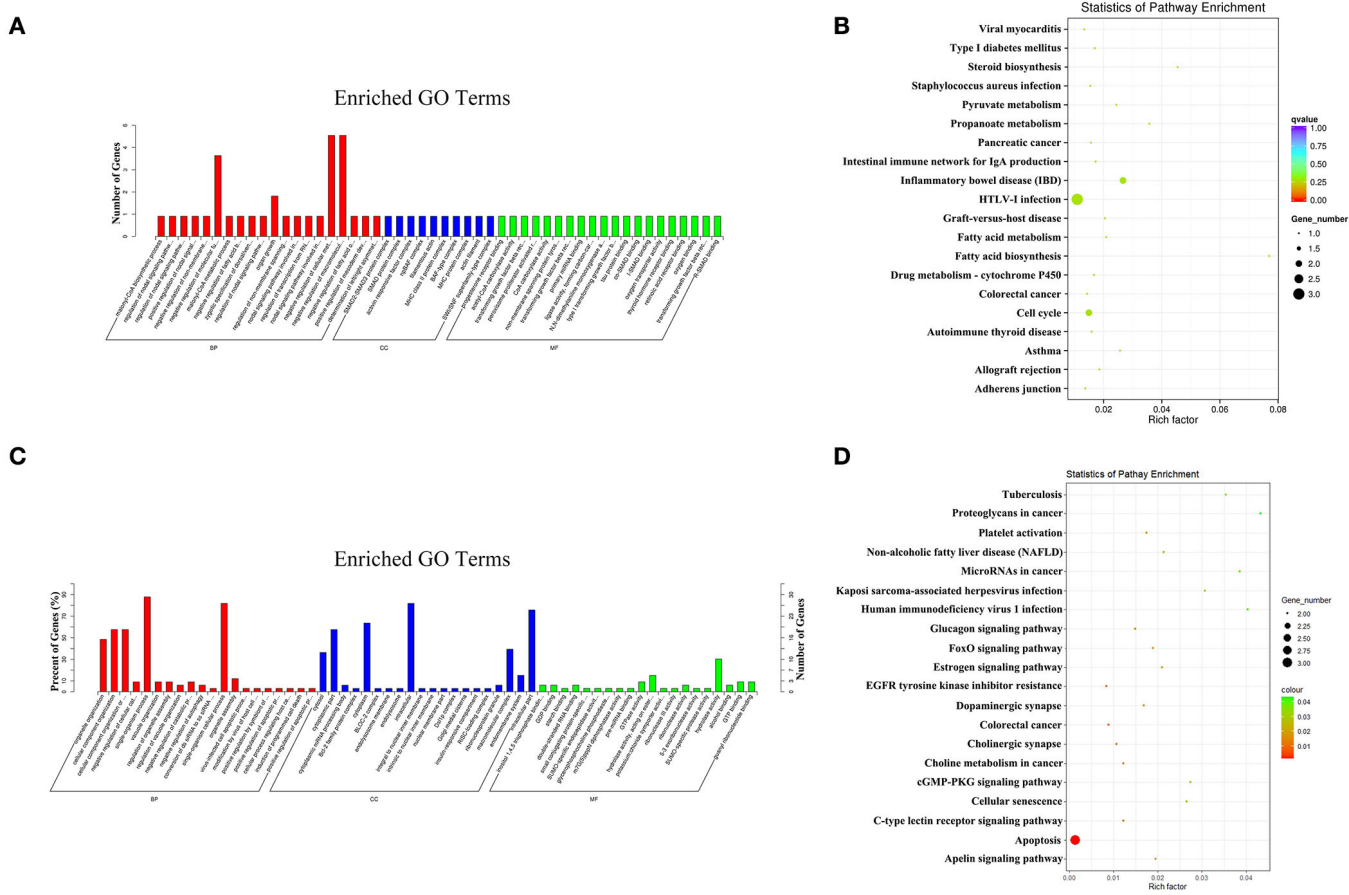
The most enriched GO terms **(A)** and KEGG pathways **(B)** of the target genes of the DE miRNAs identified in PL vs. EL comparison. The most enriched GO terms **(C)** and KEGG pathways **(D)** of the parental genes of the DE circRNAs identified in PL vs. EL comparison.

In the PL vs. EL comparison, the target genes of the DE miRNAs were significantly enriched in 76 GO terms, and the most enriched GO terms were malonyl-CoA biosynthetic process (GO: 2001295) in BP, SMAD2–SMAD3 protein complex (GO: 0071144) in CC, and progesterone receptor binding (GO: 0033142) in MF (Figure 7A). The parental genes of the DE circRNAs were significantly enriched in 72 GO terms, and the most enriched GO terms were organelle organization (GO: 0006996) in BP, cytosol (GO: 0005829) in CC, and inositol 1,4,5 trisphosphate binding (GO: 0070679) in MF (Figure 7C).

In terms of KEGG enrichment, the target genes of the DE miRNAs were significantly enriched in five KEGG pathways (Figure 7B), and the three most enriched KEGG pathways were inflammatory bowel disease (IBD, oas05321), HTLV-I infection (oas05166), and fatty acid biosynthesis (oas00061). Non KEGG pathway was significantly enriched for the parental genes of the DE circRNAs (Figure 7D), the three most enriched KEGG pathways were apoptosis (oas04210), epidermal growth factor receptor (EGFR) tyrosine kinase inhibitor resistance (oas01521), and colorectal cancer (oas05210).

### Identification of sheep lactation biomarkers using machine learning approaches

Parameters used in the present study was selected with a systematic evaluation of a range of hyperparameter values. The detailed parameter training results and biomarker identification results are given in Supplementary Table S7.

**Figure 8:**
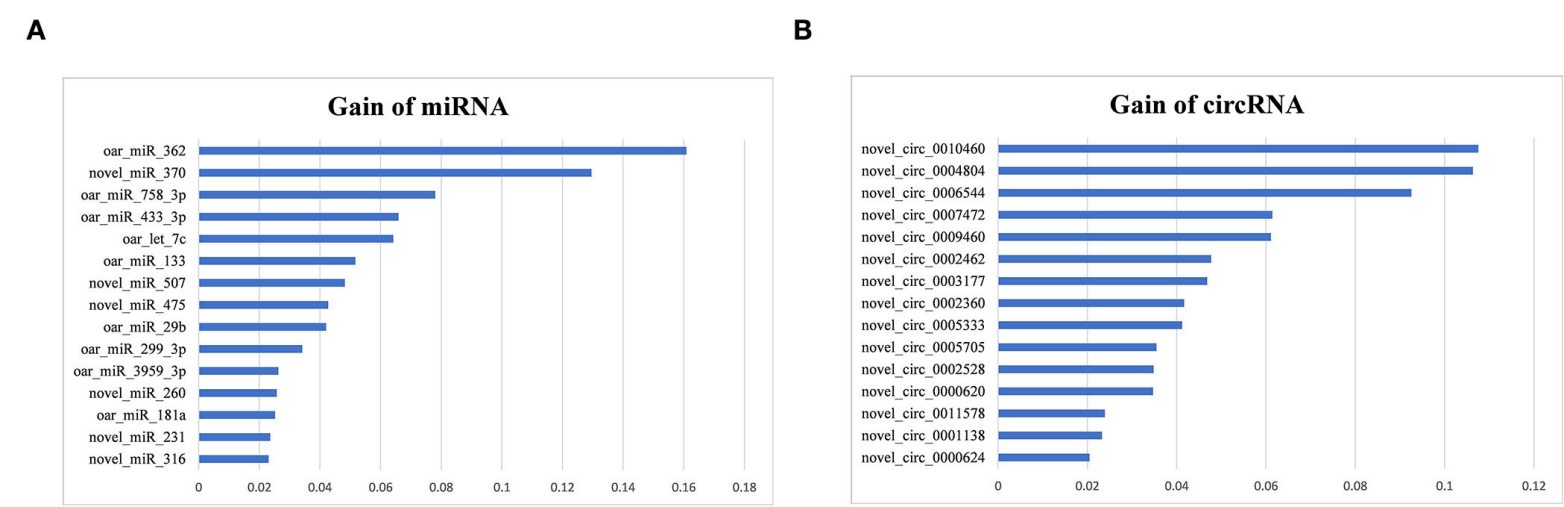
Gain values of the top miRNA **(A)** and circRNA **(B)** biomarkers of sheep lactation identified.

To identify miRNA biomarkers, 114 positive-VIM miRNAs were first selected with Random Forest, and then 38 of those miRNAs were further selected with XGBoost. Three miRNAs with the highest Gain values (Figure 8A) were oar_miR_362 (0.16), novel_miR_370 (0.13), and oar_miR_758_3p (0.08).

To identify circRNA biomarkers, 885 positive-VIM circRNAs were first selected with Random Forest, then 42 of those circRNAs were further selected with XGBoost. Three circRNAs with the highest Gain values (Figure 8B) were novel_circ_0010460 (0.11), novel_circ_0004804 (0.11), and novel_circ_0006544 (0.09).

### ceRNA network

**Figure 9:**
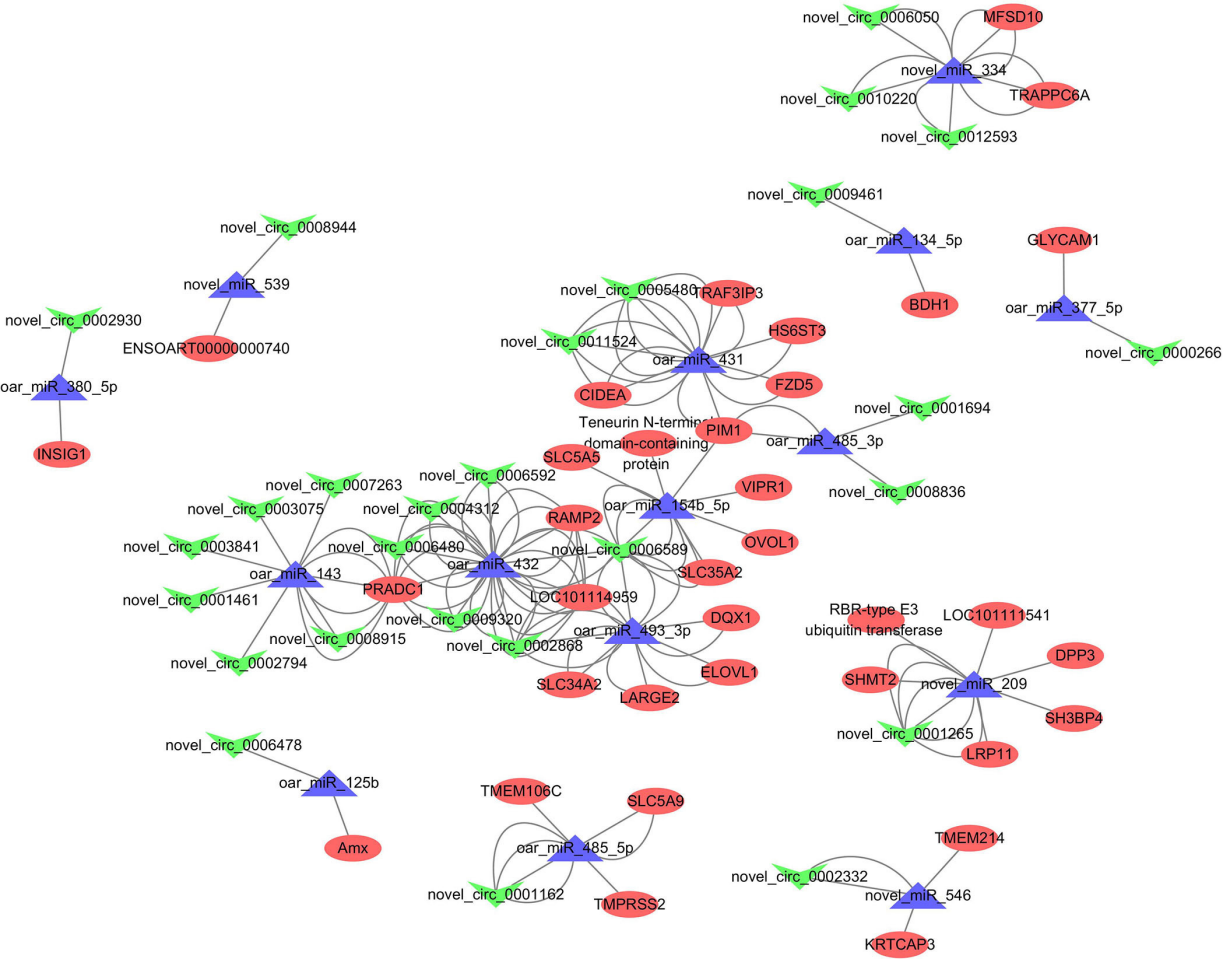
Competing endogenous RNA (ceRNA) networks, in which the “V” shape (green), triangle (purple), and circle (red) represent circRNAs, miRNAs, and messenger RNAs (mRNAs), respectively.

When we combined the results of miRanda, RNAhybrid, calculated PCC, and adjusted *p*-values, 130 miRNA–circRNA interactions and 68 miRNA–mRNA interactions were identified. ceRNA networks were then constructed based on the shared miRNAs, and we finally obtained 73 competing circRNA–miRNA–mRNA triplets containing 27 circRNAs, 15 miRNAs, and 36 mRNAs Figure 9. Within these, the most strongly connected candidates circRNA, miRNA, and mRNA were novel_circ_0006589 (13), oar_miR_432 (31), and *PRADC1* (12), respectively. Detailed results are given in Supplementary Table S8.

### Validation of RNA-seq data

**Figure 10:**
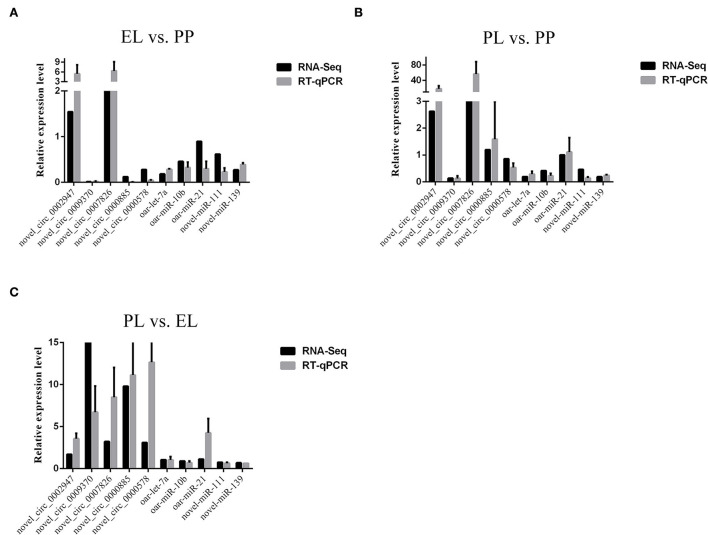
Comparisons of the results of RNA-sequencing (RNA-seq) and quantitative PCR (qPCR) analyses of selected non-coding RNAs in the EL vs. PP **(A)**, PL vs. PP **(B)**, and PL vs. EL comparisons **(C)**.

The expression levels of selected non-coding RNAs determined with RNA-seq and quantitative PCR (qPCR) are presented in Figure 10. The results show that the expression of both the selected circRNAs and miRNAs was similar in the RNA-seq and qPCR analyses, confirming the accuracy of our sequencing data.

## Discussion

Lactation is a dynamic and multifactorial process in mammary gland development (32). At the transcription level, mammary gland development is regulated by a number of genes and non-coding transcripts, including *DGAT* (33), miR-143 (34), lncRNA Neat1 (35), and circ11103 (12). In our previous study (6), we systemically investigated the expression profiles of mRNAs and lncRNAs at three key points during mammary gland development in sheep (PP, EL, and PL), and detected a number of candidate genes and lncRNAs. However, the transcriptomic mechanisms that underlie mammary gland development are not fully understood, especially the roles of non-coding transcripts. In the present study, we investigated the expression profiles of miRNAs and circRNAs in PP, EL, and PL, to determine how miRNAs and circRNAs functions in mammary gland development, and their regulatory roles in controlling the expression of lactation-related genes.

### Expression profiles of circRNAs and miRNAs

In total, 2,369 circRNAs and 272 miRNAs were annotated. Compared with a previous transcriptome study on dairy cattle (36) and other sheep breeds (31), which identified over 4,000 circRNAs in the mammary gland, remarkably fewer circRNAs were identified in the present study. A possible explanation for this discrepancy is that various animal models differ in their properties.

Among the annotated circRNAs, the most highly expressed circRNAs (according to the average TPM) were novel_circ_0011066, novel_circ_0011021, and novel_circ_0010252, whose parental genes are *SLTM, USP3*, and *SLC39A8*, respectively. It is noteworthy that *SLTM, USP3*, and *SLC39A8* are closely related to the differentiation of mammary stem cells, mammary epithelial cell cycle, and mammary gland expansion (37–39). Moreover, Ahmad showed that circ_87295 from *USP3* was highly expressed in the mammary gland of Kashmiri cattle (13), and Hao et al. showed that circ_011411 from *SLC39A8* was highly expressed in the mammary gland of small-tailed Han sheep (3). Taken together, these data suggest that these circRNAs and their parental genes function similarly in mammary gland development across species. The most highly expressed of the annotated miRNAs were oar_miR_148a, oar_miR_143, and oar_miR_30a. A previously published study demonstrated that these miRNAs are involved in mammary gland development. For example, miR_148a regulates milk triacylglycerol synthesis in goat mammary epithelial cells (40), miR_143 regulates milk fat synthesis in bovine mammary epithelial cells (41), and the miR_30 family is involved in fatty acid metabolism in the mammary gland (42). Although the specific functions of these highly expressed circRNAs and miRNAs in sheep are still largely unclear, our results suggest that they act as principal regulators during mammary gland development, as they do in other species. Of course, in-depth work is still required to confirm this proposition.

When DEseq was applied, 30, 34, and 7 DE miRNAs, and 348, 373, and 36 DE circRNAs were detected in the comparison of EL vs. PP, PL vs. PP, and PL vs. EL, respectively. Consistent with our previous study on lncRNAs and mRNAs, clearly different expression profiles of both circRNAs and miRNAs were revealed between the non-lactation period (PP) and the lactation periods (EL and PL). Our results imply that the transcriptomic status of the mammary gland during lactation may be relatively stable, in terms of both coding and non-coding transcripts. It is noteworthy that novel_circ_0010160 was found to be DE in all three comparisons. Specifically, the expression level of novel_circ_0010160 rapidly decreased from the PP to the EL and then slowly increased from EL to PL. Previous studies on *TBC1D14* (parental gene of novel_circ_0010160) demonstrated its important role in cancer cell autophagy (43, 44). This evidence suggests that novel_circ_0010160 may also function similarly in sheep MEC autophagy and would make a prime candidate for future research, especially in the non-lactation period.

When the non-lactation period and lactation periods were compared (EL vs. PP and PL vs. PP), 171 shared DE circRNAs and 16 shared DE miRNAs were identified. Of these, the most upregulated circRNA and miRNA (ranked by fold changes and adjusted *p-*value) were novel_circ_0011345 and oar_miR_148a, respectively. The parental gene of novel_circ_0011345 is feline leukemia virus subgroup C receptor-related protein 2 (*FLVCR2*). Little is known about the roles of this circRNA and its parental gene *FLVCR2* in lactation, but the strong expression of novel_circ_0011345 in the lactation period suggests that it acts as a principal regulator during mammary gland development. As mentioned earlier, miR_148a has been shown to play an important role in mammary metabolism during lactation (45), and it is highly probable that miR_148a also acts as a key regulator of sheep milk production. The most downregulated circRNA was novel_circ_0005886, whose parental gene is zinc-finger 532 (*ZNF532*), a member of the C2H2-type zinc-finger family. Previous studies conducted in mice have suggested that the expression of circRNAs from *ZNF532* is positively associated with cell apoptosis and pyroptosis (46). Considering the diverse roles of the zinc-finger family in mammary gland cell organization (47), it is therefore conceivable that novel_circ_0005886 also has certain effects on sheep milk production, probably by regulating the cellular BPs of sheep mammary epithelial cells. The most strongly downregulated miRNA, oar_miR_99a, showed markedly higher expression in PP than in lactation periods. Little is known about oar_miR_99a in mammary gland development, but a similar study conducted by Laurent et al. in the mammary glands of Prealpes-du-Sud ewes showed that miR_99a is also highly expressed in the non-lactation period (48). Collectively, these findings suggest that miR_99a is a prime candidate for future research on mammary gland development, particularly during the onset and early stage of lactation.

As noted in the abovementioned sections, small subsets of DE circRNAs and miRNAs were identified within the lactation periods. This finding raises the question of why sheep milk production varies in different lactation periods. The comparison of PL and EL identified DE circRNAs and miRNAs, which provide clues about the modification of the non-coding transcriptomic profile during lactation. The most upregulated (PL high) circRNA and miRNA were novel_circ_0001786 and oar_miR_218a, respectively. The parental gene for novel_circ_0001786 is *LSM14A*, and most research on these transcripts to date has focused on their roles in immunity (49, 50). However, their strong expression during PL, which corresponds to an intensive phase of proliferation in the mammary gland, suggests that the expression of novel_circ_0001786 and oar_miR_218a contribute to milk production in sheep, or at least some aspects of it.

The expression of the most downregulated transcripts, novel_circ_0006360 and oar-miR-181a, rapidly decreased from EL to PL. The parental gene of novel_circ_0006360 is *PCM1*, a key gene in both mammary gland development and breast cancer (51), which is related to cell proliferation and the cell cycle. Therefore, novel_circ_0006360 may act as a principal regulator in mammary gland development and may function similarly to its parental gene: *PCM1*. miR-181a, a star miRNA, has been shown to be associated with multiple mammary gland-related BPs, including milk fat biosynthesis (52), the heat stress response (53), and the mammary immune system (54). However, it is noteworthy that numerous studies have shown that the expression miR-181a is suppressed during the dry period relative to the lactation period in dairy cattle (55) and goats (56), which is inconsistent with our findings. Based on these findings, we speculate that miR-181a may play opposite roles as it does in other species, and may be involved in multiple functions that regulate mammary gland development.

### Functional enrichment of DE miRNAs and DE circRNAs

To further define the biological functions of DE miRNAs and DE circRNAs, GO and KEGG functional enrichment analyses were conducted on the target genes of DE miRNAs and the parental genes of DE circRNAs.

In comparisons between PP and lactation periods, the GO annotation showed that the target genes of DE miRNAs and the parental genes of DE circRNAs were mainly involved in cellular progress, such as intracellular organelle, membrane-bounded organelle, and the endosome. In comparisons of lactation periods (PL vs. EL), the target genes of DE miRNAs and the parental genes of DE circRNAs were significantly enriched in metabolic processes in the mammary gland during lactation, such as negative regulation of single-organism process, cellular metabolic process, and macromolecule metabolic process. Consistent with previous findings in the dairy goat (57), these results suggest that the identified miRNAs and circRNAs differentially expressed in the non-lactation and lactation periods mainly contributed to diverse lactation-related cellular processes, especially in the organelles. Meanwhile, miRNAs and circRNAs differentially expressed in different lactation periods were found to regulate metabolism-related processes.

The KEGG enrichment analysis showed that the target genes of DE miRNAs and the parental genes of DE circRNAs identified in the comparison between PP and lactation periods were mainly enriched in ECM–receptor interactions and the AMPK signaling pathway. ECM–receptor interactions are important components of focal adhesions, which are involved in the migration of mammary epithelial cells during lactation (58). The AMPK signaling pathway is important in cellular energy sensing and in the regulation of glucose supply and utilization in the lactating mammary gland (59). Collectively, the DE miRNA and DE circRNAs identified here may act as regulators of diverse cellular processes and stimulate the development of mammary glands. Similar to the results of the GO enrichment analysis, the target genes of DE miRNAs and the parental genes of DE circRNAs when the lactation periods were compared were significantly enriched in metabolism- and cellular biology-related KEGG pathways, including fatty acid metabolism, the cell cycle, and apoptosis. Our results identified the regulatory roles of these DE miRNAs and DE circRNAs in the cell cycle and fatty acid metabolism, which may be responsible for differences in milk yield and milk components in the EL and PL periods.

### Identification of sheep lactation biomarkers using machine learning approach

Previously, we compared the classification accuracy of several decision tree-based machine learning approaches (Random Forest, XGBoost, and RX) and DE transcript identification methods (edgeR and *t*-test) in subsets of transcriptomic data. The results showed that a method combining Random Forest and XGBoost (RX) outperformed the other four methods (Random Forest, XGBoost, *t*-test, and edgeR) with the highest classification accuracy and showed biological value in the prediction of multiple traits such as feed efficiency (26) and *Escherichia coli* infection (60).

For these reasons, RX was used to identify the biomarkers of sheep lactation in this study. Of all identified biomarkers, oar_miR_362 and novel_circ_0010460 outperformed all candidate transcripts with the highest “Gain” value, indicating the importance of oar_miR_362 and novel_circ_0010460 in distinguishing the different lactation periods. Although the specific roles of oar_miR_362 and novel_circ_0010460 in lactation are still unknown, their high gain values show their power to distinguish different lactation periods, and are also evidence for the critical roles of oar_miR_362 and novel_circ_0010460 in mammary gland development. The algorithm underlying the decision tree also demonstrates the strong interactivity between the top biomarkers and the other identified candidate biomarkers, indicating additional roles for oar_miR_362 and novel_circ_0010460 in sheep lactation. Of course, systematic functional verification is still required to clarify the biological roles of oar_miR_362 and novel_circ_0010460 in sheep lactation.

### ceRNA network

In recent decades, many studies have demonstrated that circRNAs can function as competing endogenous RNAs, sharing miRNA recognition elements, and regulating target gene expression at different stages of mammary gland development (10, 36). To clarify the circRNA-related ceRNA crosstalk underlying mammary gland development, we constructed ceRNA networks of circRNA–miRNA–mRNA triplets.

A total of 73 competing circRNA–miRNA–mRNA triplets were identified, within which several key regulators were found, including miR-143 [lipid droplet formation, (41)], miR-125b [mammary inflammatory response (61)], SLC family members [cell cycle (62)] and *OVOL1* [mammary epithelial–mesenchymal transition, (63)]. The most connected regulators in the ceRNA networks were novel_circ_0006589, oar_miR_432, and *PRADC1*. Previous studies have shown that these transcripts have been widely investigated in cancers (64, 65), whereas little is known about their functions in the mammary gland. Our findings provide basic evidence that these most strongly connected transcripts in the network are probably the key ceRNA regulators in sheep mammary gland development. Further research is required to confirm our hypothesis.

## Conclusion

In conclusion, this study has characterized the expression profile of miRNAs and circRNAs in the mammary gland of Hu sheep during different lactation periods for the first time. By combining a DE analysis, functional enrichment, machine learning prediction, and a ceRNA network analysis, we identified subsets of candidate miRNAs (e.g., oar_miR_148a, oar_miR_362, and oar_miR_432) and circRNAs (e.g., novel_circ_0011066, novel_circ_0010460, and novel_circ_0006589) involved in the development of the sheep mammary gland. Consequently, our study provides a foundation for future research on the non-coding molecular mechanisms underlying sheep lactation.

## Data availability statement

The datasets presented in this study can be found in online repositories. The names of the repository/repositories and accession number(s) can be found below: https://www.ncbi.nlm.nih.gov/, PRJNA853925 and https://www.ncbi.nlm.nih.gov/, PRJNA854211.

## Ethics statement

The animal study was reviewed and approved by Experimental Animal Welfare and Ethical of Institute of Animal Science, Yangzhou University.

## Author contributions

Conceptualization: WC, XL, XC, ZY, SW, and WS. Data curation and formal analysis: WC, XG, and XL. Supervision and funding acquisition: WS. Writing—original draft: WC. Writing—review and editing: WC and WS. All authors read and approved the final manuscript.

## Funding

This work was supported by the National Natural Science Foundation of China-CGIAR (32061143036), National Natural Science Foundation of China (31872333 and 32172689), Major New Varieties of Agricultural Projects in Jiangsu province (PZCZ201739), The Projects of Domesticated Animals Platform of the Ministry of Science, Key Research and Development Plan (modern agriculture) in Jiangsu province (BE2018354), Jiangsu Agricultural Science and Technology Innovation Fund [CX182003], Jiangshu 333 Distinguished Talents Project Foundation [(2022) 2-323], and Jiangsu Postgraduate Research and Innovation Program (KYCX21_3260).

## Conflict of interest

The authors declare that the research was conducted in the absence of any commercial or financial relationships that could be construed as a potential conflict of interest.

## Publisher's note

All claims expressed in this article are solely those of the authors and do not necessarily represent those of their affiliated organizations, or those of the publisher, the editors and the reviewers. Any product that may be evaluated in this article, or claim that may be made by its manufacturer, is not guaranteed or endorsed by the publisher.

## Supplementary material

Supplementary Figure S1 Pearson correction of miRNAs and circRNAs between different samples.

Supplementary Table S1 Detailed information of version and parameters of used software.

Supplementary Table S2 Sequence and designed primers of selected micro ribonucleic acids (miRNAs) and circular RNAs (circRNAs).

Supplementary Table S3 An overview of miRNA/circRNA sequencing data.

Supplementary Table S4 Detailed information of annotated miRNAs and circRNAs.

Supplementary Table S5 Differentially expressed (DE) miRNAs and circRNAs.

Supplementary Table S6 Detailed enrichment results of the target genes of the DE miRNAs and parental genes of the DE circRNAs.

Supplementary Table S7 Detailed results of RX.

Supplementary Table S8 Information of circRNA–miRNA–mRNA competing endogenous RNA (ceRNA) network.
