# Supplementary figures and images for: Non-coding transcriptomic profiles in the sheep mammary gland during different lactation periods
Source: Front Vet Sci. 2022 Nov 8;9:983562. doi: 10.3389/fvets.2022.983562 (PMC9679157; doi:10.3389/fvets.2022.983562)

**A**

Pearson correlation between samples

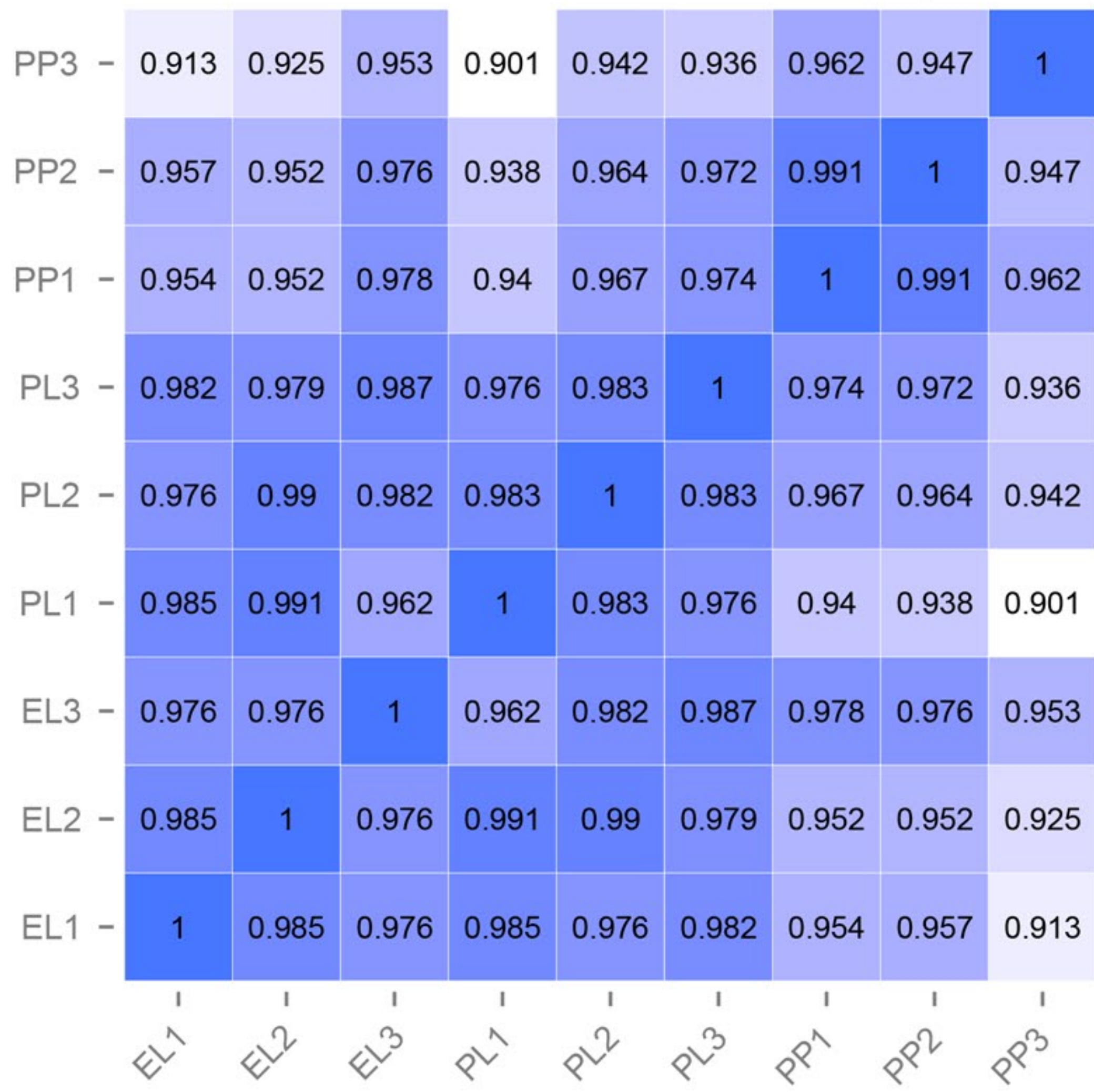**miRNA****B**

Pearson correlation between samples

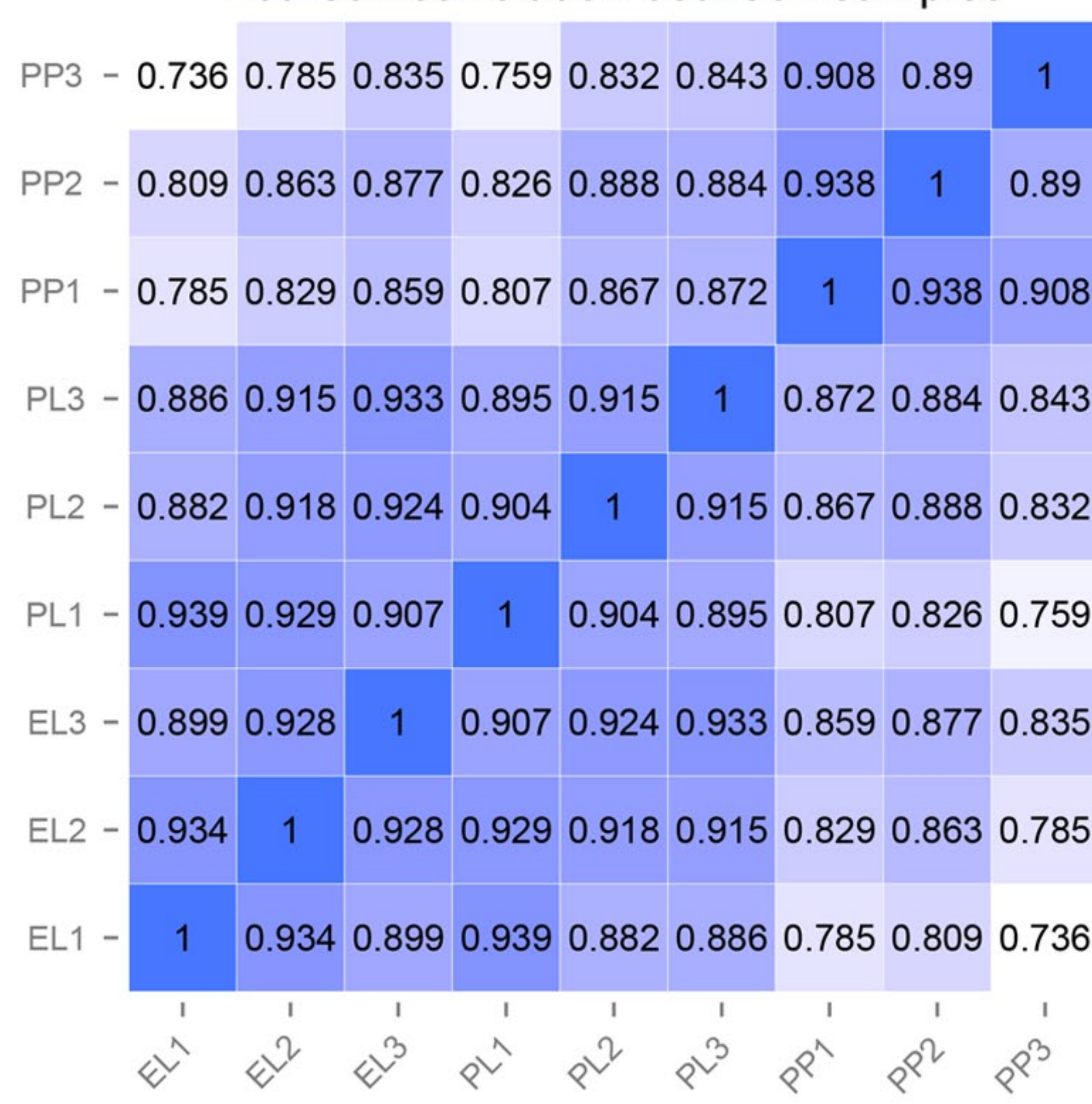**circRNA**

Supplement: Supplementary Figure S1 — Pearson correction of miRNAs and circRNAs between different samples. [file Data_Sheet_1.PDF]
